# Reviewing Federated Machine Learning and Its Use in Diseases Prediction

**DOI:** 10.3390/s23042112

**Published:** 2023-02-13

**Authors:** Mohammad Moshawrab, Mehdi Adda, Abdenour Bouzouane, Hussein Ibrahim, Ali Raad

**Affiliations:** 1 Département de Mathématiques, Informatique et Génie, Université du Québec à Rimouski, 300 Allée des Ursulines, Rimouski, QC G5L 3A1, Canada; 2 Département d’Informatique et de Mathématique, Université du Québec à Chicoutimi, 555 Boulevard de l’Université, Chicoutimi, QC G7H 2B1, Canada; 3 Institut Technologique de Maintenance Industrielle, 175 Rue de la Vérendrye, Sept-Îles, QC G4R 5B7, Canada; 4 Faculty of Arts & Sciences, Islamic University of Lebanon, Wardaniyeh P.O. Box 30014, Lebanon

**Keywords:** federated machine learning, federated learning, privacy preservation, aggregation algorithms, diseases prediction, cardiovascular diseases, diabetes, cancer, smart wearables, smart health

## Abstract

Machine learning (ML) has succeeded in improving our daily routines by enabling automation and improved decision making in a variety of industries such as healthcare, finance, and transportation, resulting in increased efficiency and production. However, the development and widespread use of this technology has been significantly hampered by concerns about data privacy, confidentiality, and sensitivity, particularly in healthcare and finance. The “data hunger” of ML describes how additional data can increase performance and accuracy, which is why this question arises. Federated learning (FL) has emerged as a technology that helps solve the privacy problem by eliminating the need to send data to a primary server and collect it where it is processed and the model is trained. To maintain privacy and improve model performance, FL shares parameters rather than data during training, in contrast to the typical ML practice of sending user data during model development. Although FL is still in its infancy, there are already applications in various industries such as healthcare, finance, transportation, and others. In addition, 32% of companies have implemented or plan to implement federated learning in the next 12–24 months, according to the latest figures from KPMG, which forecasts an increase in investment in this area from USD 107 million in 2020 to USD 538 million in 2025. In this context, this article reviews federated learning, describes it technically, differentiates it from other technologies, and discusses current FL aggregation algorithms. It also discusses the use of FL in the diagnosis of cardiovascular disease, diabetes, and cancer. Finally, the problems hindering progress in this area and future strategies to overcome these limitations are discussed in detail.

## 1. Introduction

Artificial intelligence (AI) is a rapidly advancing technology that is increasingly being integrated into various industries and aspects of daily life, leading to significant changes and advancements in the way we live and work. This truth is obvious and can be seen with one’s own eyes; no evidence is needed to prove it. Ever since Alan Turing, considered the father of theoretical computer science and artificial intelligence, asked their famous question, “Can computers think?” [1], artificial intelligence has become a broad field of research. Despite the fact that AI has been researched for a long time, there is no single definition for this field. The authors in [2] defined it as a set of tools and techniques that use principles and devices from various fields such as computation, mathematics, logic, and biology to address the problems of realizing, modeling, and mimicking human intelligence and cognitive processes, while the authors in [3] defined it as programs that, in an arbitrary world, will cope no worse than a human.

Machine learning (ML), a derivative of AI, allows computers to “learn” from training data and expand their knowledge over time without being explicitly programmed. Machine learning algorithms attempt to find patterns in data and learn from them to make their own predictions. In short, machine learning algorithms and models learn through experience. Traditionally, a computer program is developed by engineers and given a set of instructions that enable it to turn incoming data into its intended output. ML, by contrast, designs the program to learn with little or no human interaction and to expand its knowledge over time. The remarkable success of ML, as well as its enormous potential in classification and regression problems and its ability to use both supervised and unsupervised learning techniques, have made it attractive to researchers in many fields. Later studies revealed the variety of applications of ML that can be observed in the field such as:

- E-commerce and product recommendations [4,5];
- Image, speech and pattern recognition [4,5];
- User behavior analytics and context-aware smartphone applications [4,5];
- Healthcare services [6,7,8];
- Traffic prediction and transportation [4,9];
- Internet of Things (IoT) and smart cities [9];
- Cybersecurity and threat intelligence [10];
- Natural language processing and sentiment analysis [11];
- Sustainable agriculture [12];
- Industrial applications [13].

### 1.1. Machine Learning under The Scope: Challenges

**Figure 1:**
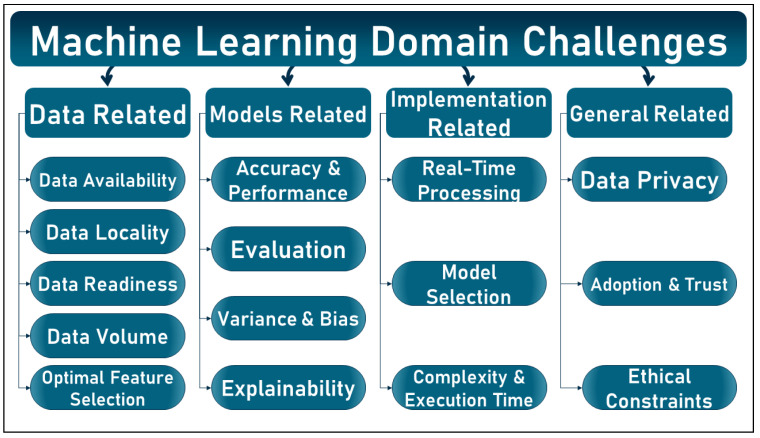
Machine Learning Domain Challenges.

Accurate results in classification or regression are increasingly encouraging the incorporation of these techniques into areas of daily life. The feasibility of using AI tools, and in particular ML, has been demonstrated by the high performance they offer and the possibility of implementing them in different domains. However, ML still suffers from several challenges that are extensively described and discussed in the literature. However, these challenges are not classified into a single taxonomy, but grouped according to different aspects. In this section, the common challenges are presented under a proposed taxonomy based on data-related, model-related, implementation-related, and other general aspects. In addition, these challenges are illustrated and summarized in Figure 1 below.

#### 1.1.1. Data Related Challenges

Machine learning algorithms are typically implemented in a known pipeline consisting of data collection, preprocessing, exploration, model selection, training, evaluation, and deployment. Data, which constitute the main component of these algorithms, can present various challenges, such as [14,15]:

- Data availability and accessibility: to train a model, one must have the necessary data, which may not be available on the spot or may be available but inaccessible for various reasons;
- Data locality (data islands): in the real world, data are scattered in different and unrelated entities called “data islands.” Due to different regulations and laws, data related to the same subject and available on different data islands cannot be accessed for use and analysis;
- Data readiness: even if data are available and accessible, several aspects should be considered, such as:
  - Data heterogeneity: available data may have different characteristics or be composed of different forms. For example, health data for the same patient may be available in different forms, such as medical images, reports, videos, and structured data. The ability to deal with such heterogeneity is a challenging task;
  - Noise and signal artifacts: due to the interaction between data acquisition instruments and other electrical devices, data can be poisoned by noisy attributes that affect the overall results of ML models;
  - Missing data: data collected by measuring devices may be incomplete for various reasons;
  - Classes imbalance: in classification problems, the data collected for one group may dominate the data collected for other groups, affecting the learning of the smart model.
- Data volume: is the amount, size, and scope of the data. In the context of ML, size can be defined either vertically by the number of records or samples in a dataset or horizontally by the number of features or attributes it contains. Data volume presents several challenges, such as:
  - Course of dimensionality: dimensionality describes the number of features or attributes that are present in a dataset. Increasing dimensionality can have a negative impact on model performance;
  - Bonferroni principle [16]: the Bonferroni principle states that when searching for a particular type of event in a given set of data, the probability of finding that event is high. Therefore, the accuracy of a ML model subject to the Bonferroni principle may be compromised.
- Feature representation and selection: the performance of ML models heavily depends on the choice of data representation or features, so selecting the optimal features will definitely improve the overall model performance.

#### 1.1.2. Models Related Challenges:

In addition to the challenges posed by the data, the models themselves can present researchers with various problems, such as [17,18]:

- Accuracy and performance: achieving the highest accuracy for ML models remains the main goal for researchers from various fields, and the highest accuracy will lead to the highest adoption and integration of this technology;
- Model evaluation: evaluating an ML model can be challenging, especially when traditional performance metrics such as accuracy, precision, and recall do not reflect a model’s feasibility;
- Variance and bias: where variance is the variability of the model prediction for a given data point or a value indicating the spread of our data, and bias is the difference between the average prediction of our model and the correct value we are trying to predict. ML models are susceptible to variance and bias, which can affect their performance, results, and confidence;
- Explainability: some of the ML models, especially deep learning models, are known by their black box identity. The lack of explanations of how they work can have a negative impact on trust in these models, even when high accuracies are achieved.

#### 1.1.3. Implementation Related Challenges:

Assuming that the obstacles in the data and models have been overcome, implementing the models of ML can be a challenging task due to various obstacles such as [19,20]:

- Real-time processing: ML models are created and trained with available data. However, fitting these models to real-time problems presents several challenges;
- Model selection: different models can produce different results even for the same problems. For example, support vector machines (SVM) and logistic regression (LR) can lead to different results, even when working with the same data at the same point in time. Thus, selecting the optimal model and tuning its parameters are not easy tasks;
- Execution time and complexity: due to the complexity of the data or models, multiple preprocessing steps, and many other reasons, ML models can require enormous computing power and long execution times.

#### 1.1.4. General Challenges:

Finally, other challenges besides technical aspects can be mentioned in this section, such as [17,18]:

- User data privacy and confidentiality: which is one of the most critical issues in the field of ML. Users tend not to share their data for various reasons, which affects the availability of the data and jeopardizes the entire ML cycle;
- User technology adoption and engagement: due to privacy issues, unclear results, lack of explanation, and other reasons, users may not accept ML being integrated into their daily routine, or even accept its results;
- Ethical constraints: various ethical constraints posed by ML have been widely discussed in the literature, such as control and morality, model ownership, environmental impact, and many others.

### 1.2. Privacy Challenge: Federated Machine Learning Motivation

The challenges in machine learning and its derivatives have been thoroughly studied, and researchers are trying to find answers to all of them without focusing on just one. Nevertheless, the workflow of ML mainly consists of data acquisition and preprocessing, feature engineering, model training, model evaluation, and model deployment. The structure of the workflow reflects the importance of data in ML. The performance of ML models heavily depends on the availability of data. Although achieving highly accurate models depends on the technical structure of the models themselves, the cleanliness and readiness of the data, the optimal feature selection, and many other aspects, it is well known that the availability of more data to train the models increases their accuracy [14,15]. However, in the real world, data collection is a big challenge, if not the biggest, in developing ML models for several reasons, most importantly privacy and confidentiality.

Not only individuals, but also society, governments, and organizations are strengthening the protection of data privacy and security. In this regard, several regulations and laws were enacted, such as the European Union’s General Data Protection Regulation (GDPR) [21], China’s Cyber Security Law of the People’s Republic of China [22], the General Principles of the Civil Law of the People’s Republic of China [23], the PDPA in Singapore [24], and hundreds of principles legislated around the world. While these regulations help protect private information, they pose new challenges to the ML field by making it more difficult to collect data to train models, which in turn makes it more difficult to improve the accuracy of model performance and to personalize those models. Consequently, data privacy and confidentiality are not a stand-alone challenges, but also trigger other challenges for ML, such as data availability, performance, personalization, and thus trust and acceptance.

#### Overcoming Privacy Challenges

The criticality of privacy has been a hot research topic for years, pushing to find different solutions to protect the information exchanged by subjects. To this end, various privacy algorithms were proposed, such as encrypting data before exchange through various algorithms such as differential privacy [25], k-order anonymity [26], homomorphic encryption [27], and other methods. However, these methods were not able to provide definitive and unbreakable solutions, as several attacks have been observed in ML such as the model inversion attack [28] and the membership inference attack [29], which are able to derive raw data by accessing the model.

Recently, Google proposed a new concept in the machine learning domain known as “federated machine learning” or “federated learning” [30]. The main concept behind FL is to eliminate the exchange of user data between peripherals. FL is a type of collaborative distributed/decentralized ML privacy-preserving technology where a model is trained without the need to transfer data from the edges to a central server, but models are sent to peripherals to be trained on local data, and then sent back to a central aggregation server to generate the global model without knowing the embedded data.

Federated learning has proven to be a great solution to user privacy issues, opening the door to collecting more data to train ML models and improve their accuracy and efficiency. Moreover, FL enables training models with data from different entities known as data islands and merging the knowledge into a global trained model, which increases the efficiency of the models. In addition, FL enabled the handling of heterogeneous data scattered in different data spaces with different characteristics, and facilitated the so-called “learning transfer” where models can share their knowledge without transferring users’ private data. Nevertheless, FL is still in its infancy and is still vulnerable to various challenges.

### 1.3. Machine Learning and Healthcare

The development of information and communication tools, in parallel with the emergence of artificial intelligence and its branches such as ML and DL has produced effective solutions to health challenges. Moreover, AI is considered the most promising technology for improving healthcare services, as it can be applied to almost all areas of medicine and will revolutionize healthcare delivery to patients and populations. This tremendous contribution is not due to magic, but to AI’s data processing capabilities that surpass those of humans, especially in terms of its ability to perform large calculations in a short period of time. Given the promise, initiatives to use AI as a solution to healthcare problems have recently significantly expanded, with the number of AI healthcare applications exceeding thousands in the last decade [31,32].

AI is playing an increasingly important role in healthcare and has the potential to revolutionize the way healthcare professionals diagnose, treat, and monitor patients. One of the most important ways in which AI can be used in healthcare is to analyze large amounts of medical data. By using machine learning algorithms to identify patterns and trends in these data, AI can help medical professionals make more accurate diagnoses, predict which patients are at risk of developing certain diseases, and develop more personalized treatment plans [33]. AI can also be used to monitor patients’ health and vital signs in real-time, and to alert medical professionals to potential problems. This can be particularly useful for patients with chronic conditions who need close monitoring to avoid complications. For example, using AI in smart wearables, a person’s heart rate and blood pressure can be continuously monitored and the data analyzed to detect the early signs of cardiovascular diseases (CVDs), as shown in [34]. In addition, smart wearables equipped with sensors and machine learning algorithms can play a critical role in detecting and monitoring diabetes by continuously tracking and analyzing biometric data such as blood glucose levels, heart rate, and activity levels, enabling early detection and intervention [35]. In addition, the potential of smart wearables and machine learning models in detecting fatigue in the workplace has been shown to be highly feasible, contributing to disease prevention [36]. Overall, AI has the potential to significantly improve the quality of healthcare for patients and make healthcare more efficient and cost-effective. However, it also raises ethical and legal issues that need to be addressed for the successful implementation of AI in healthcare.

With healthcare being of critical importance, the performance of ML in healthcare needs to be enhanced. Increasing this performance requires using the latest techniques and overcoming any barriers that may impede progress. The barriers to the development of the use of ML in healthcare are the same for all ML implementations in all diseases and correspond to the previously described problems. Therefore, potential solutions that can help promote the use of ML will lead to improved applications in these areas.

### 1.4. Outline and Main Contributions of This Article

In this article, FL and its use in disease prediction and diagnosis have been studied. To achieve this goal, this article explores this topic in depth in the following sections. In Section 2, FL is discussed from various perspectives, including technical perspectives, aggregation algorithms, and others. Then, in Section 3, the use of Federated learning technology in detecting and predicting various diseases is presented by listing the state-of-the-art in each area and discussing the implementations mentioned in the literature. Later, in Section 4, the challenges that hinder the progress in this field are discussed and therefore some future perspectives that could help in overcoming these challenges are proposed. In this context, this article attempts to answer the following questions:

- What is federated machine learning?
- What are the motivations for this technology?
- What are the technical perspectives on which FL is based?
- What taxonomy can be used to classify FL algorithms and techniques?
- What are the differences between FL, traditional ML (including deep learning), distributed and decentralized ML, and federated database systems?
- What are the existing FL aggregation algorithms and what is the contribution of each?
- What are the available FL frameworks?

The topic of federated learning has been a hot and trending topic in recent years. As a result, dozens if not hundreds of studies have already addressed this topic, with a large number of these studies reviewing federated learning. However, none of the articles proposed an inclusive and full taxonomy for FL, or even compared FL to classical ML, decentralized ML and federated database systems. Furthermore, the federated aggregation algorithms were not reviewed with any of the previous studies. Furthermore, the use of FL in diseases prediction such as CVDs and diabetes were not reviewed. Consequently, this article proposes several new ideas, contributing to the body of FL knowledge by:

- Proposing a novel and comprehensive taxonomy that classifies FL into the maximum number of possible categories;
- Establishing clear and precise boundaries to distinguish between FL, traditional ML, distributed and decentralized ML, and federated database systems;
- Discussing existing aggregation algorithms in FL and evaluation of the contributions of each to the field;
- Reviewing and discussing the state of the art of FL in diagnosing:
  - Cardiovascular disease;
  - Diabetes;
  - Cancer.
- Presenting the challenges faced by FL and the possible future perspectives that can be pursued to increase the efficiency of the technology.

## 2. Federated Learning

Artificial intelligence and its derivatives, such as machine learning and deep learning, are gaining attraction and confidence in a variety of fields. For example, deep learning surpassed human performance in the game of Go, where AlphaGo and AlphaGo Zero achieved superhuman feats by beating the world champions of the game. However, the high accuracy achieved by these models required that they be trained on data that spanned 29 million records [37,38]. This underscores the description of such technologies as “data hungry,” with the need to improve the accuracy of the models requiring larger datasets. This is undoubtedly the case not only in gaming, but also in other sectors such as education, industry, healthcare, and others. Moreover, this is not the only problem that hinders the development of ML and DL. With the development of ICT tools and especially mobile networks, data collection has become easier and larger datasets are being obtained. However, an urgent problem that requires effective solutions is the privacy and security of data, with the disclosure of information about individuals never being a minor issue and recently attracting the attention of both governments and researchers [39,40].

### 2.1. Overview and Definition(s)

The increasing efficiency of artificial intelligence tools is leading them to be used in various areas of life. However, the challenges faced by these technologies lead researchers to always look for appropriate solutions, which is why federated learning, or what is sometimes called federated machine learning, was found.

#### 2.1.1. Data Islands and Privacy Dilemma: Concept behind FL

**Figure 2:**
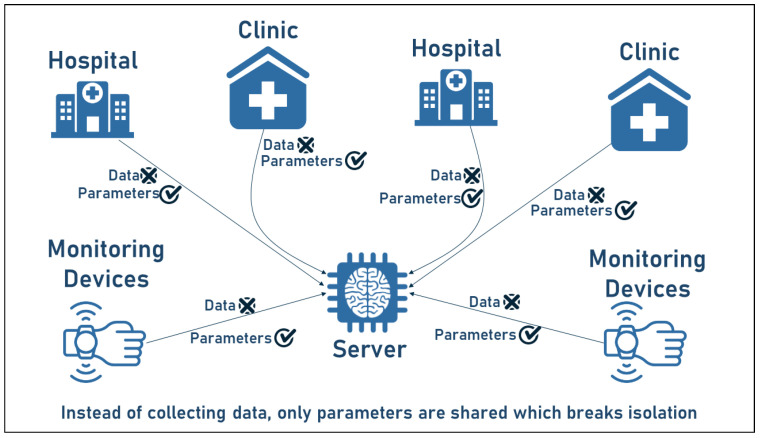
Data islands concept illustrated by medical entities.

The ability to collect and analyze large amounts of data has recently made great strides, especially with the development of communication tools and AI methods. However, data are collected in what are known as “data islands.” Data islands are defined as foundations, institutions, individuals, or other entities where data are collected and stored [41,42]. To improve the performance of AI models, the idea of the centralized server is pursued, with the common method being to collect data in a centralized repository and perform unified processing, cleaning, and modeling. For example, a patient’s health data scattered across different hospitals, clinics, or health centers have the greatest potential when analyzed together [43]. However, privacy regulations and restrictions, as well as data heterogeneity, limit the ability to collect and simultaneously analyze such data. Consequently, the search for solutions to the data islands and privacy dilemma has attracted the attention of researchers worldwide and was the motivation for the concept of federated learning [44]. In Figure 2, the concept of data islands is illustrated by showing how medical data may be stored in different institutions and cannot be shared due to the sensitivity of the health data. Instead, the parameters are shared with the FL server as shown in the figure.

#### 2.1.2. Motivations behind FL Concept

The critical importance of data protection has led to the development of various protection algorithms aimed at protecting data through encryption or other methods, but they have failed to provide an inevitable solution against attacks. Moreover, the data annotation in some fields, such as the medical industry, relies on the knowledge of professionals, resulting in a rarity of valid data that are detrimental to industrial development. Accordingly, the need to deal with private data or data scattered in islands while maintaining their privacy is the main motivation behind the concept of federated learning [45]. The fact that private and confidential data available in scattered sources are more usable for ML models than those centralized on the server provides FL with the opportunity for collaboration between these data sources to improve the accuracy of ML models [46]. Because the data can be analyzed without having to be transferred to a central server, FL helps address the challenges mentioned earlier. The FL architecture, communication methods, security mechanisms, and other factors allow the model to be trained on edge devices, the data islands, by sending them the model itself, rather than collecting and aggregating the data in a centralized space [47]. In other words, instead of aggregating training data from different sources, FL enables the training of the shared global model using a central server, while keeping the data at their main sources of origin [48]. This not only preserves data privacy, but also reduces data transfer costs by limiting the transfer to only the necessary parameters rather than the entire datasets. This also allows dealing with a scalable number of devices, ranging from ten to ten million [49,50]. All in all, FL is an emerging and promising technology that helps one solve the ML challenges by preserving data privacy, increasing the model performance, reducing the data transfer costs, improving scalability, and more. Therefore, it has the potential to challenge the prevailing ML paradigm [51,52].

#### 2.1.3. FL Definition(s)

Federated learning was originally introduced by Google in 2016, where it was used in Google Keyboard to predict users’ text input on tens of thousands of Android devices without transferring data from the devices to central servers [30]. However, the authors in [43] claim that the term FL was introduced before and that its core idea is distributed deep learning, such as the privacy-protected deep learning system proposed in [52]. Although it is still considered a new concept, it is increasingly attracting researchers’ attention, and its definition can be found in various places in the literature. For example, the authors in [42,45] define it by explaining how it works, mentioning that federated learning is a type of collaborative distributed/decentralized machine learning technology where privacy is maintained and a model is trained without the need to transfer data from the edges to a central server, but instead weight updates are sent to a central aggregation server to build the global model. A statistical definition is given in both [41,44], where FL is defined as follows:

“Define N data owners {F1, …FN}, all of whom wish to train a machine learning model by consolidating their respective data {D1, …DN}. A conventional method is to put all data together and use D = Di ∪ … ∪ DN to train a model MSUM. A federated learning system is a learning process in which the data owners collaboratively train a model MFED, in which process any data owner Fi does not expose its data Di to others. In addition, the accuracy of MFED, denoted as VFED should be very close to the performance of MSUM, VSUM. Formally, let δ be a non-negative real number, if |VFED−VSUM|<δ. We say the federated learning algorithm has δ-accuracy loss.”

### 2.2. FL Technical Inspection

The potential for federated learning lies in the architecture upon which it is built. To understand this structure, it is necessary to study the various aspects of this technology and its various parts, which will be presented in this section.

#### 2.2.1. Underlying Architecture

Federated learning is a collaborative decentralized approach of machine learning where data are analyzed by the model without being transmitted from the edges to the central server, which acts as an aggregator. This is made possible by the architecture behind this technology. The technical architecture of FL consists of the three main components: the parties, the manager, and the communication framework, which are discussed below [41,42,44]:

- Parties: are also referred to as customers, users, or individuals, and are the data owners and beneficiaries of FL. They are indicated by:
  - Hardware specifications such as storage, processing, and power capacities;
  - Scalability and stability;
  - Data distribution.
- Manager: known as a server or aggregator, is the high-performance central server that acts as a model aggregator rather than a data collector;
- Communication–computation framework: the computation handles the model training and the communication handles the exchange of model parameters between the parties and the manager. Several frameworks were developed to manage the relationships between different FL entities, which are discussed in detail later;

In the various available frameworks for communication computation, the steps taken in the application of FL differ but they share a common basic concept which is:

- The parties federally train their own model using their local data without sharing it;
- The global model is updated by the locally trained models;
- The global model is then shared with the different parties/data owners;
- The above steps are repeated until the global model achieves the desired performance.

#### 2.2.2. FL Communication-Computation Frameworks

The different FL communication–computation frameworks are due to the different centralized concepts. Currently, there are two FL concepts: centralized managers and decentralized managers. Each of these concepts manages communication between parties differently, where [46]:

- Centralized design (client-server architecture): in this approach, data flow is often asymmetric, with the manager aggregating information from the parties and sending them back to the updated model. In addition, communication between the manager and the parties can be synchronous or asynchronous;
- Decentralized design (peer-to-peer architecture): In this approach, communication is performed between the parties themselves without the need for a central manager. This allows each party to directly update the global parameters.

**Figure 3:**
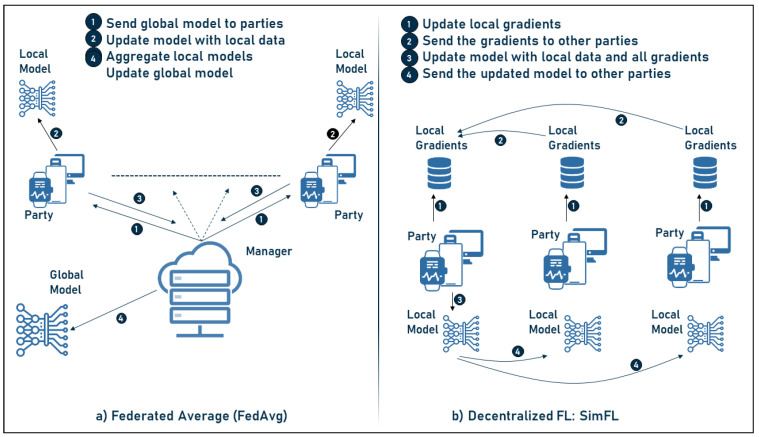
Communication–computation frameworks.

The above concepts are currently implemented in various FL frameworks which will be discussed later. Two popular FL architectures are mentioned below: the centralized federated average (FedAvg) [30] and the decentralized FL framework [53], which are discussed and explained below as well as shown in Figure 3:

- Federated average learning, which is the basis of FL and is determined in the following steps:
  - The manager sends the model to the parties involved;
  - The parties train the received model with their local data;
  - The updated models are sent back to the manager;
  - The above steps are repeated until the model achieves the desired performance.
- Decentralized federated learning SimFL, where no central manager/server is required. In this framework, the following steps are applied:
  - The parties first update the gradients of their local data;
  - Then, the gradients are sent to a selected party;
  - Next, the selected party uses its local data and the gradients to update the model;
  - Then, the model is sent to all other parties;
  - To ensure fairness and to use the data from the different parties, each party is selected to update the model for approximately the same number of rounds and the above steps are therefore repeated until the final model is reached.

### 2.3. Federated Learning Taxonomy

The different ways of applying federated learning have contributed to the creation of numerous classifications within this technology, which can be considered differently according to the different subdivision bases or points of view. In light of this, the study of the literature in FL concludes to subdivide it based on six approaches, which are listed below and explained in this section:

- Data partitioning;
- Machine learning model;
- Privacy mechanism;
- Communication architecture;
- Scale of federation;
- Motivation of federation.

#### 2.3.1. Data Partitioning

Federated learning provides the ability to train models without the need to collect data from edge devices. In addition, in the FL environment, a device’s local storage of data samples (pictures, documents, etc.) is considered its sample space. On the other hand, the feature space is the collection of characteristics used to characterize the data points, often expressed as a vector with a large number of dimensions. This set of characteristics may be put to use in a wide range of classification and regression applications. FL is able to develop a model that can efficiently aggregate information from the various sample and feature spaces, which are typically dispersed throughout the parties (clients, users, etc.).

**Figure 4:**
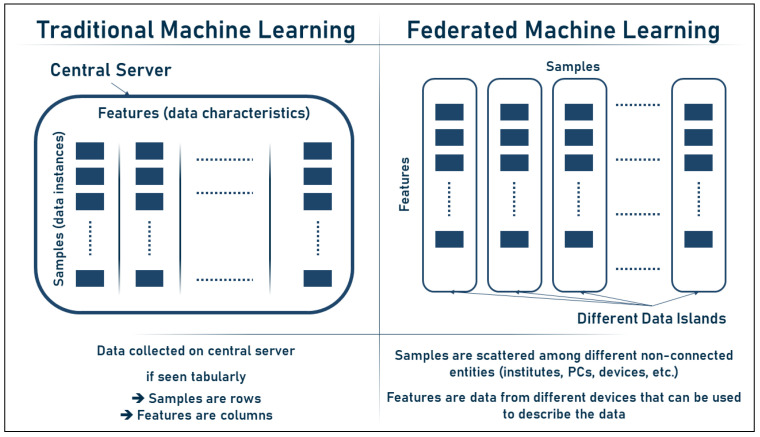
Samples vs. features in traditional and federated ML.

Depending on the data structure and point of view, the samples and features in federated machine learning (FL) may be seen as rows or columns. Traditional machine learning uses a table-like data structure with rows representing samples and columns representing features; in FL, however, the samples are generally dispersed over numerous devices or locations, leading to a lack of unified data structure. If this is the case, we may think of the samples as columns and the features as rows, with each feature being shared across all devices. Finally, the representation is determined by the nature of the issue and the FL technique used. Figure 4 below describes the difference between features and samples in both traditional ML and federated ML.

In this context, the different forms of data partitioning in federated learning environments form three categories that are described below [41,42,44].

- Horizontal FL: also known as sample-based federated learning, and is the case when the data on the parties share the same feature space but differ in the samples. In other words, in horizontal FL partitioning, the datasets are partitioned horizontally (by parties), and then the part of the data that have the same features but the parties are not exactly the same is taken out for training. It is therefore characterized by the following:
  - Is the most commonly used data partitioning strategy in implementations of FL;
  - Is suitable to increase the sample size;
  - Can train the local models using their local data with the same architecture, since these data share the same feature space;
  - Simplifies the update of the global model by averaging all local models.
- Vertical FL: also known as feature-based learning, when the data share the same or similar sample space (parties) but differ in the feature space (data). In other words, in vertical FL partitioning, the dataset is split vertically (by features), then part of the data where the parties are the same but the features are not exactly the same are taken out for training:
  - Which is challenging in terms of implementation;
  - Which makes it more complex to update a global model by averaging because the data may not be similar between parties;
  - Which has much more room for improvement to be applied in more complicated ML approaches.
- Federated transfer learning: this is the case when the datasets scattered between the parties differ not only in the samples but also in the feature space. In this partitioning method, the data are not segmented, but the learning is transferred to overcome the lack of data or tags. Therefore, it is characterized by:
  - Being an effective way to protect both data security and user privacy while breaking the boundaries of data islands;
  - Enabling the transfer of knowledge from one domain to another for better learning outcomes;
  - Offering plenty of room for growth to make it more flexible with different data structures;
  - Triggering the issue of communication efficiency.

**Figure 5:**
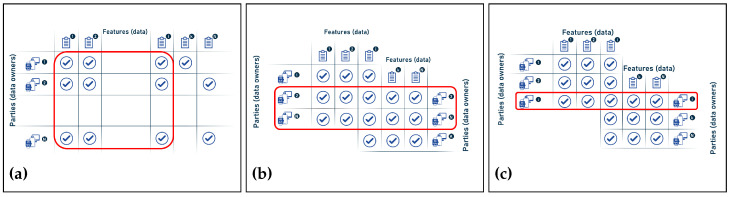
(**a**) Horizontal FL; (**b**) vertical FL; and (**c**) federated transfer learning.

Furthermore, Figure 5 below illustrates the three categories of federated machine learning divided by the type of data.

In Table 1, the differences between the alternate groups of FL, classified based on the type of data, are summarized.

#### 2.3.2. Machine Learning Models

Federated learning was created to overcome problems with machine learning algorithms. Therefore, it is of great interest to train a modern ML model for a specific task. Researchers have worked diligently to develop new models or reinvent existing models to fit the federated learning architecture. For example, the ML models used in FL include but are not limited to: [41,42,44]:

- Linear models: support vector machines, linear regression, ridge regression, lasso regression, among others;
- Decision tree: gradient boosting, decision trees, random forests, among others;
- Neural networks: convolutional neural networks, multi-layer perceptron, deep neural networks, and others.

#### 2.3.3. Privacy Mechanism

It is clear that the main goal behind the development of FL technology is to protect the privacy of the data of individuals, organizations, and companies participating in the machine learning process. The main concept to preserve this privacy is that the parties involved do not share their data with other entities, but only exchange some model parameters. However, these parameters may still reveal sensitive information about the data. FL was exposed to several attacks that may occur at any stage of the process of FL, including the inputs, the learning process, and the learned model [54]. In the list below, several attacks are discussed and detailed based on the model stage targeted by the Machine Learning attack [46]:

- Inputs: During this phase, malicious parties can perform “data poisoning attacks” [55,56,57], in which the labels of the training samples with a particular class are changed so that the final model performs poorly on that class;
- Learning process: during this process, parties can perform “model poisoning attacks” [58,59] or Byzantine fault [60,61] to upload some designed model parameters at the local model level. Such attacks can negatively affect the accuracy of the learning process due to the poisoned local updates;
- The learned model: once the learned model is published, it is exposed to attacks such as model inversion attack [28] and membership inference attack [29] and others. Such attacks can potentially infer raw data by accessing the model. For example, they can determine whether a particular dataset was used in the training process. Finally, inference attacks can also be performed in the FL manager learning process, where the server has access to the parties’ local updates.

To overcome such problems and achieve the goals, various approaches such as model aggregation, cryptographic methods, and differential privacy are used in Federated Learning systems. These techniques help avoid the risk of attacks and backdoors and are described below [41,42,43]:

- Model aggregation: is one of the most common privacy preserving mechanisms in FL systems and the main concept behind the FL technique, where the global model is trained by aggregating the model parameters of all parties without sharing the original data in the training process;
- Cryptographic methods: In this approach, the parties must encrypt their messages before sending them to the manager or other parties, work with the encrypted messages, and decrypt the encrypted output to obtain the final result. In this context, various algorithms have been used in FL systems, such as:
  - Homomorphic encryption [39]: Users can compute and process the encrypted data without revealing the original data, and at the same time the user decrypted the processed data with the key, which is exactly the expected result. However, due to the additional encryption/decryption operations, homomorphic encryption incurs extremely high computational overhead;
  - Secure multiparty computation (SMC) [62]: in this algorithm, the server is guaranteed to learn the parties’ inputs only in their entirety. However, SMC does not provide any confidentiality guarantee for the final model, which is still vulnerable to inference and model inversion attacks and can also be a reason for additional computational overhead.
- Differential privacy [63]: is a new definition of privacy in which the final results of the model are insensitive to the changes of a particular dataset by minimizing the impact of a single dataset on the computation of the results. This method has been proven successful for data poisoning attacks, but may not be usable for model poisoning attacks.

#### 2.3.4. Methods for Resolving Heterogeneity

The different equipment of the parties involved in the FL system and the diversity of the data stored in them can have a negative impact on the efficiency of the overall learning process. To solve the problems caused by this heterogeneity, four types of distractions are used in FL implementations [41]:

- Asynchronous communication: the synchronous scheme can be easily disrupted by the diversity of devices. Therefore, asynchronous communication can help resolve this diversity;
- Device sampling: limiting the use of a party/device to only the necessary iterations, not necessarily participating in every single iteration;
- Fault-tolerant mechanism: in an environment with multiple working participants, the failure of one participant can affect the performance of the entire environment. A fault-tolerant mechanism helps prevent the entire system from collapsing if one of the parties fails;
- Model heterogeneity: is used to resolve data heterogeneity and includes three strategies:
  - Each individual party has its own model;
  - A global model that is suitable for all parties;
  - Relevant learning models for tasks.

#### 2.3.5. Communication Architecture

Following the various client/server approaches taken in FL systems, there are two main categories in communication architecture, which are [46]:

- Centralized design: this assumes the existence of a central server that aggregates the local models trained by the parties and sends them back for updating. Communication between the manager and the local parties can be synchronous or asynchronous;
- Decentralized design: in this approach, communication is between the parties, and each can directly update the global model without the need for a central aggregation manager.

#### 2.3.6. Scale of Federation

Federated learning can be classified into two groups based on the scale of federation, namely: cross-silo FLS and cross-device FLS [42,46]. These two approaches differ in the number of parties and the amount of data stored in each party, where [64]:

- Cross-silo FL: this approach is used when the participating parties are fewer in number, have relatively large amounts of data, have relatively high computational power, and are available for all rounds of learning. This approach is best suited when the participants are organizations or computers;
- Cross-Device FL: in contrast, the number of parties involved in the learning process is relatively large, they have a small amount of data, and are equipped with relatively low computing power. This approach is best suited when the participants are mobile devices.

#### 2.3.7. Federation Motivation

Finally, the reasons for using FL systems can be categorized as follows [46]:

- Regulations: where laws restrict the sharing of private information between different companies, such as the GDPR, Chinese laws, or PDPA or other laws;
- Incentives: where FL is motivated by a desire to develop services.

The various categories of federated learning that may be obtained from grouping various points of view are outlined below in Table 2 along with a summary of the advantages associated with each category.

### 2.4. Federated Learning: Borderlines

Federated learning is the result of the accumulation of technological improvements in machine learning. Motivated by privacy preservation, inspired by the concept of distributed computing, and executed by advanced communication technologies, FL has become an efficient and feasible technology. In this section, we highlight the limitations of FL systems to differentiate them from traditional and previous ML technologies.

#### 2.4.1. FL vs. Classic ML

Both FL and classical ML aim to optimize the learning goal. However, they differ in the architecture of their models. Since the classical ML can be implemented in both centralized and distributed approaches, this section compares FL only with the centralized classical ML, while the comparison with the distributed ML is performed in the next section. Centralized classical ML is the concept where data characterized by the same features are collected from different users on a central server where they are then processed and analyzed. In this context, the two concepts are compared using [47]:

- Motivation: classical ML focuses on the learning goal, while FL focuses on both the learning goal and privacy;
- Data identity: in classical ML, user data are described as independently and identically distributed (IID), while in FL, it is possible to deal with unbalanced non-IID data coming from different parties, be it individuals or institutions;
- Centralization: in the classical ML, all data and computations are centralized around one server, while FL provides both centralized and distributed server architecture;
- Data access: in the classic ML, the central server has full access to the user data, while this is not the case in FL;
- Communication and data transfer: in classic ML, all the user data are fully transmitted to the central server, while in FL, only minimal parameters or trained models are exchanged.

#### 2.4.2. FL vs. Distributed and Decentralized ML

The architecture of the FL system is based on the concept of distributed computing. Therefore, FL is considered a collaborative distributed learning technology. On the other hand, distributed classical ML is the concept that collects data characterized by the same features from different users on more than one central server where they are processed and analyzed. Thus, the concept of distributed classical ML is to distribute the data analysis tasks to multiple servers instead of just one. Thus, it can be said that distributed classical ML models are trained using the same methodology as centralized ML models, except that they are trained separately on multiple servers. In this context, the two concepts are compared using [41,42,43]:

- Motivation: in distributed classical ML, the main goal is to accelerate the processing phase, while in FL, both privacy and processing phases are targeted;
- Data identity: in the distributed classical ML, the data are described as IID records, while in FL, it is unbalanced non-IID records due to heterogeneity;
- Centralization: in the distributed classical ML, no central server is included in the architecture, while in FL, both centralization and distribution are provided;
- Data access: in the distributed classic ML, the data are distributed among several servers, but the global model still has access to the user data and, moreover, some servers can have access to all the data of a user at a given time;
- Communication and data transmission: in distributed classical ML, all user data are transmitted to the network of servers, while in FL, only minimal parameters or trained models are exchanged.

#### 2.4.3. FL vs. Federated Database System

Federated database systems (FDSs) [65] are systems that are able to combine multiple database entities and manage them as one overall system. This concept was proposed to achieve integration between multiple independent databases. Moreover, it can manage heterogeneous databases distributed among different storage units. Moreover, FDS focuses on basic operations such as insert, delete, update, and other database operations. In this context, the two concepts are compared using [44,65]:

- Motivation: in FDBS, the main goal is to perform database operations over diverse and independent databases, while the main goal of FL is to process heterogeneous and independent databases to learn from data;
- Data identity: both can support non-IID databases;
- Centralization: both support the decentralization of database storage, but in FDBS, the processing is handled by a central server;
- Data access: in FDBS, unlike FL, the processing server has access to all data;
- Communication and data transfer: in FDBS all data are transferred in contrast to FL.

**Figure 6:**
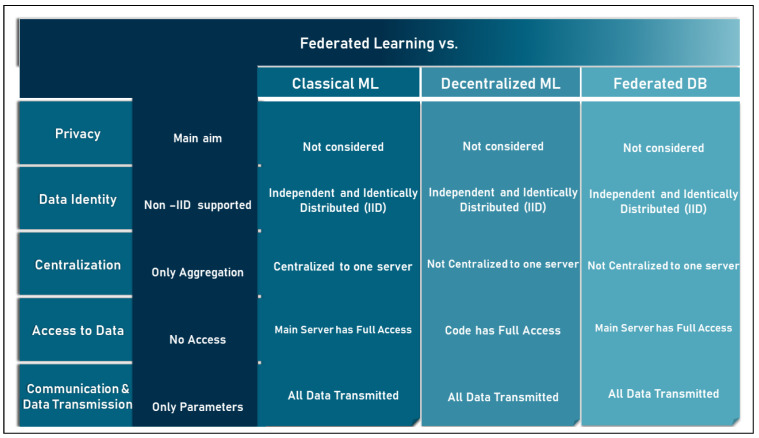
Borderlines between FL, ML, decentralized ML and federated DB.

The boundaries between federated ML and classical machine learning, distributed and decentralized machine learning, and the federated database are shown in Figure 6 below.

### 2.5. FL Aggregation Algorithms: State of the Art

The first implementation of federated learning was proposed by Google to train Android keyboards to predict text input [30]. Despite its success in training machine learning models without the need to collect user data, the performance of FedAVG is poorly understood and encounters a number of problems and drawbacks, as discussed in [66]. These drawbacks can be summarized below:

- Performance issues:
  - Suffering from ‘client-drift’ and convergence;
  - Tuning difficulty;
  - High communication and computation cost;
  - Significant variability in systems characteristics on each network device;
  - Existence of non-identically distributed data across the network;
  - Heterogeneity of devices, users and network channels;
  - Sensitivity to local models;
  - Scalability issues.
- Security and privacy issues: FL is still under the risk of several breaching attacks such as:
  - Poisoning attacks;
  - Inference attacks;
  - Backdoor attacks.

Therefore, there was a great need to improve the performance of the federated learning FedAvg aggregation algorithm to overcome its drawbacks. In this context, several implementations have been carried out in the last 5 years. Given the diversity of challenges in this area, researchers are continuously investing in developing or improving FL aggregation algorithms. To this end, there are twenty-seven aggregation algorithms in the literature to date. These algorithms are listed in Table 3 below. An in-depth analysis of these algorithms can summarize the areas to which they contribute in the following list, which is also detailed in the table:

- Improving model aggregation;
- Reducing convergence;
- Handling heterogeneity;
- Enhancing security;
- Reducing communication and computations cost;
- Handling users’ failures (fault tolerance);
- Boosting learning quality;
- Supporting scalability, personalization, and generalization.

**Figure 7:**
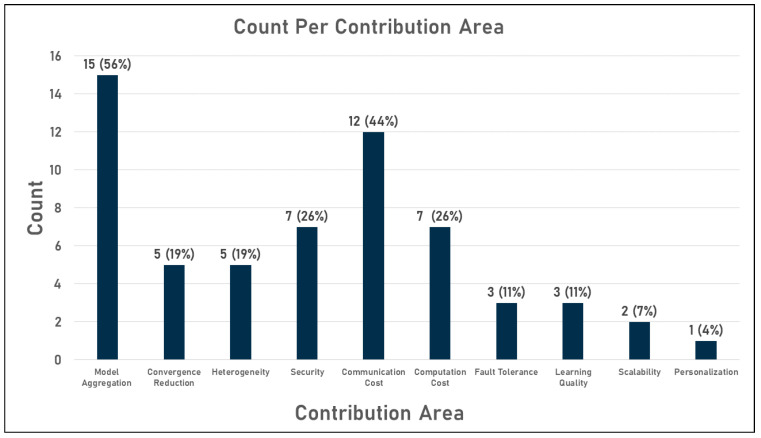
Aggregation algorithms count per contribution area.

However, the achievements of previous federated learning aggregation algorithms have mainly focused on the aggregation itself or on reducing communication costs. The other contribution areas have been less studied. For example, among the 27 algorithms mentioned, 15 targeted global model aggregation and 12 targeted communication cost reduction, while only three targeted learning quality improvement and only one targeted personalization. This distribution is shown in the diagram in Figure 7 below.

Analysis of the distribution of implementations per contribution domain shows that the state of the art in federated learning algorithms has produced a number of robust aggregation algorithms that are also acceptable from the point of view of reduced communication costs. However, from a security point of view, all the presented implementations focused on only one type of attack, namely the Byzantine attack. Other attacks have not been extensively covered in the literature, raising the question of how robust the available methods are against attacks such as reversal attacks, which are the main concern of FL, where attackers can detect users’ private data based on the local trained model exchanged within the network. In addition, few efforts have been made to improve the learning quality of the models from FL, which in turn raises the question of the extent to which the accuracy of the traditional algorithms from ML is comparable to that of the models from FL. Finally, personalization has only been investigated in a single study, as shown in the table and chart.

### 2.6. FL Available Frameworks/Platforms

Despite its novelty, federated learning has been a popular topic in the research community. The increasing interest in this field assisted in having several frameworks or platforms that implement FL. Some of those frameworks are [65,92,93]:

- Tensorflow federated (TFF) algorithm [94]: an open source framework for experimenting with FL that enables developers to experiment with novel FL algorithms as well as simulating existing ones on their data;
- Federated AI technology enabler (FATE) [95]: relies on homomorphic encryption and supports a range of FL architectures and secure computation algorithms including logistic regression, tree-based algorithms, neural networks and transfer learning;
- PySyft [96]: developed by OpenMined and decouples private data from model training using federated learning, differential privacy and multiparty computation;
- Tensor/IO [97]: a lightweight cross-platform library for on-device machine learning, bringing the power of TensorFlow and TensorFlow Lite to iOS, Android, and React native applications;
- Tensorflow encrypted: provides an interface similar to that of TensorFlow and aims to make the technology readily available without requiring the user to be an expert in ML, cryptography, distributed systems, and high-performance computing;
- CoMind: built on top of TensorFlow and provides high-level APIs for implementing FL and FedAvg specifically;
- Horovod: based on the open message passing interface (MPI) and works on top of popular deep learning frameworks, such as TensorFlow and PyTorch;
- LEAF benchmark: is a modular benchmarking framework for machine learning in federated settings, with applications in FL, multi-task learning, meta-learning, and on-device learning aiming to capture the reality, obstacles, and intricacies of practical FL environments.

### 2.7. Training and Evaluation of Federated Learning Algorithms

FL is known as a privacy-preserving technology, where the data are not transferred to nor collected at a central server to allow model training. However, when training a federated machine learning model, updates are aggregated from multiple decentralized nodes: each node trains a local model on its own data and then shares the model updates with other nodes, allowing the global model to converge towards a stable solution while protecting the privacy and security of the individual data points. Additionally, there exist, in fact, norms and standards that may be used to evaluate federated machine learning algorithms. However, due to the fact that federated machine learning is still a relatively new field, these norms and standards are still in the process of developing. These norms include, but are not limited to [30,94,95,96,97]:

- Model accuracy: in the case of FL, model accuracy is a frequent parameter used to assess performance. Precision, recall, F1-score, and area under the curve (AUC) are various ways in which a model’s efficacy may be evaluated;
- Communication overhead: since communication delays might have a negative effect on the efficiency of a federated machine learning system, it is crucial to keep this in mind. The length of time spent communicating, the number of times messages need to be sent back and forth, and the overall quantity of data communicated are all indicators of communication overhead;
- Convergence speed: the speed with which a model reaches a stable solution is known as its convergence speed. Since the models in federated machine learning need to be trained across numerous participants, this is a crucial factor to take into account;
- Privacy: since the data are being shared across several parties, privacy and security are crucial concerns in federated machine learning. Examples of privacy and security standards include data encryption, differential privacy, and safe multiparty computing.

These are some of the norms and standards that are used to assess federated machine learning algorithms. However, given that the area of study is still developing, new norms and standards may appear as the technology progresses.

## 3. Federated Learning in Action

Federated machine learning is emerging as a privacy-friendly technology that is expected to boost the performance of machine learning algorithms by enabling more data analytics. The ability to analyze more data or even instances with heterogeneous architectures will help increase the accuracy of smart models and thus increase their adoption in various domains. This is already demonstrated in the literature where FL is already being used in various domains such as healthcare, transportation, Internet of Things, and others [43,50,51].

### 3.1. FL: Areas of Implementation

Federated learning was initially used to improve the text prediction service for Android Google keyboards. However, its success and efficiency motivated its implementation in other domains. As an innovative modeling mechanism that allows training a global model with heterogeneous data from different parties without compromising user data privacy and security, FL has demonstrated its feasibility for training models that classic ML models do not allow due to factors such as intellectual property rights, privacy regulations, data confidentiality, statistical heterogeneity, and others. In addition, several FL implementations have been performed in different domains such as:

- Smart healthcare: due to the sensitive nature of healthcare data, FL is a promising solution to improve the ML healthcare service while maintaining privacy [51,98];
- Smart retail: the ability to gather knowledge from different institutions enables the smart retail sector to thrive by analyzing data scattered on different islands [43];
- Transportation: FL helps improve autonomous driving decisions by training vehicles with data from different geographic locations that enable accurate learning [43,99];
- Natural language processing (NLP): with the ability to handle heterogeneous data, FL is a good choice to improve the performance of NLP models [43,100];
- Finance: the banking sector is one of the biggest beneficiaries of FL, where the data of customers scattered in different institutions can be analyzed to assess credit risk [43,50].

### 3.2. Federated Learning and Disease Prediction

In addition, federated learning has the potential to play an important role in healthcare by enabling the training of models using distributed and decentralized health data [51,93,98]. This can help protect patient privacy while enabling the creation of more accurate and personalized models and the analysis of more data, as long as privacy is maintained. Federated ML can also enable the training of models with data that are difficult to obtain and consolidate, such as data from under-served or rural areas. In addition, ML can help eliminate healthcare data islands by enabling data sharing and analysis across multiple organizations. In addition, FL has increased its efficiency in learning from data that are distributed across multiple sites and cannot be combined into a single dataset, or when data reside in multiple clinical systems [101]. In summary, FL can significantly improve the quality of healthcare by making it more data-driven and personalized [93,98,101].

#### 3.2.1. Federated ML and Cardiovascular Diseases: State-of-the-Art

Cardiovascular diseases, which comprise the deadliest diseases, claimed 18.6 million lives worldwide in 2019, accounting for 32% of global mortality. For this reason, researchers in the field of machine learning have been addressing the issue of cardiovascular diseases and trying to find more feasible solutions that can help in predicting these diseases to reduce their deadly impact. Several implementations have been performed in the literature to predict CVDs or heart-related information, whether using smart wearables equipped with smart machine learning models [34] or using only machine learning models as shown in [102].

However, with the advent of federated learning, it became possible to analyze data from diverse and heterogeneous sources, supporting the accuracy and feasibility of applying FL algorithms in cardiology. Consequently, FL has been considered in several implementations in the treatment of heart disease. For example, the authors in [103] were the first to apply FL in the field of cardiovascular disease. They analyzed various electronic health records (EHRs) to predict the hospitalizations of patients with heart disease in a given year based on their medical history described in the EHRs. To this end, they developed a federated optimization scheme (cPDS) to solve the sparse support vector machine (sSVM) problem and used the Boston Medical Center electronic health records to train and test their model. In addition to maintaining privacy, their model proved to scale well, and its performance was measured by the area under curve (AUC), which reached as high as 0.78.

In addition, the authors implemented a regression model in [104] to predict heart rate using federated learning. They used Polar smartwatches to collect their own data, which were analyzed using FL sequential Bayesian and empirical Bayes-based hierarchical Bayesian models. The former model was proposed to work based on a centralized FL architecture, while the latter provides an alternative decentralized but more scalable method from the perspective of a hierarchical Bayes model. They succeeded in creating a privacy-friendly and scalable model that predicted heart rate with high accuracy. Similarly, in [105], the authors implemented a time-series-to-time-series generative adversarial network (T2T-GAN), which is a centralized FL model based on LSTM, to predict blood pressure. Their study was performed using the “Cuff-Less Blood Pressure” estimation, an open source dataset available in the Kaggle datastore [106] for training and the “College of Queensland vital signs dataset” [107] for testing. In addition to the novelty of their model, they were able to maintain privacy and predict blood pressure with high accuracy.

In addition, the study [108] was performed to predict the presence of cardiovascular disease. With the goal of developing a personalized privacy-preserving model and reducing the difference between global and local data, a novel feature alignment model was developed to predict the presence of various cardiac arrhythmias. They analyzed electrocardiography (ECG) recordings from their privately collected data and their classification model achieved 87.85% accuracy. Similarly, in [109], the authors created a classification model to predict the cardiovascular risks. They analyzed the Nursing Electronic Learning Laboratory (NeLL) EHR data using a sequential pattern mining (SPM)-based framework. They created both centralized and decentralized models that could predict risk with high accuracy while protecting patient privacy.

In the same context, [110] proposed a cardiovascular arrhythmia prediction model based on federated learning. The authors built a centralized federated transfer learning and explainable 1D convolutional neural network (CNN) trained with the MIT-BIH arrhythmia database [111]. They succeeded in preserving privacy, increasing explainability, reducing communication costs, and creating a personalized model with up to 98.9% arrhythmia prediction accuracy.

Finally, in [112], the authors developed a 3D CNN for predicting hypertrophic cardiomyopathy with FL. Their centralized FL model was trained with the M&M [113] and ACDC challenges [114] datasets consisting of cardiovascular magnetic resonance images. Their model preserved privacy and achieved a performance of 0.89 AUC. The following Table 4 summarizes and presents the federated learning implementations performed with FL.

#### 3.2.2. Federated ML and Diabetes: State-of-the-Art

In addition to its role in predicting cardiovascular diseases, federated learning has also been used in diabetes detection. According to recent figures from the World Health Organization (WHO), diabetes affects approximately 422 million people worldwide, most of whom live in low and middle-income countries, and 1.5 million deaths are directly attributable to it each year. Most frustrating, however, is the fact that both the incidence and prevalence of diabetes have substantially increased in recent decades [115]. The criticality of these diseases and the increase in their numbers require innovative solutions to help manage these situations. In this context, several implementations of federated learning have already been carried out.

Additionally, in [116], the authors evaluated the effectiveness of federated neural network-based retinal microvasculature segmentation and classification of referable diabetic retinopathy (RDR) using optical coherence tomography (OCT) and OCT angiography (OCTA). For this purpose, several datasets were used, including SFU prototype swept-source OCTA, RTVue XR Avanti (OptoVue, Inc.), Angioplex (Carl Zeiss Meditec), and PLEX Elite 9000 (Carl Zeiss Meditec). The obtained results show that FL achieves comparable performance to conventional DL models while maintaining data confidentiality.

In addition, the authors of [117] developed a decentralized, privacy-protected, FL algorithm to identify individuals at high risk of developing diabetes-related problems. In their experiments, they trained and evaluated models using the “Health Facts EMR Data” dataset from Cerner. The results showed that FL can be used not only to maintain privacy but also to address issues such as class-imbalance when using real-world clinical data. In addition, FL showed similar performance to the gold standard of centralized learning, and the use of class-balancing strategies improved performance across all cohorts. In addition, in [118], the authors proposed the use of deep learning models for the diagnosis of diabetes, also known as the Diabetes Management Control System (DMCS). The system can predict patients’ glucose levels at each evaluation time point, while the classification model was designed to identify anomalous data points using a convolutional neural network (CNN) and a multilayer perceptron model (MLP). Considering the sensitive nature of patient physiological data contained in the datasets, the authors developed independent learning (IL) and federated learning to protect the privacy of user data. However, the dataset used to train and evaluate the proposed models was generated by a simulator. The results of their study show that the FL method has a higher retrieval rate (≥98.69%) than the IL method (≤97.87%). In addition, the FL-CNN model performed better than the MLP model with a recall value of 99.24% compared to 98.69% for the former and the latter, respectively.

Furthermore, in [119], the authors investigated the privacy threat of gradient inversion attacks to reconstruct identifiable retinal fundus images during diabetic retinopathy classification training with federated learning. Despite the fact that the primary goal of the research is privacy-related, the authors conducted their evaluation using the fine-grained annotated diabetic retinopathy (FGADR) dataset [120], which allows for the advanced exploration of DR diagnosis. The results show that the reconstructed images matched the respective baseline images with an accuracy level of 72.0%. In addition, the authors proposed an FL-based model for predicting diabetes in [121]. The experimental results showed that federated learning helps to overcome data isolation phenomenon, also known as data islands, between healthcare institutes, and successfully collects patient data from different facilities, which can not only improve the accuracy of the trained model but also successfully protect patient privacy. Furthermore, in [122], the authors investigated the use of federated learning to detect diabetic retinopathy and non-DR images. To this end, they created three models, including standard, FedAVG, and FedProx, and evaluated their models with five publicly available diabetic retinopathy datasets, including EyePACS [123], Messidor [124], IDRID [125], APTOS [126], and College of Auckland (UoA) [127]. The three models achieved accuracies of 92.19%, 90.07%, and 85.81%, respectively.

The aforementioned implementations of federated learning in the detection of diabetes. In FL, the model can be developed using data from different healthcare facilities without requiring a facility to provide its entire dataset, improving the generalizability of the model while maintaining data confidentiality. The state of the art in the use of federated learning in diabetes discussed in this section is summarized in Table 5:

#### 3.2.3. Federated ML and Cancer: State-of-the-Art

Differently speaking, cancer, which is the disease characterized by the uncontrolled multiplication and spread of aberrant cells throughout the body, is of particular interest to federated learning researchers. This disease is known to be a leading cause of death worldwide, responsible for approximately 10 million deaths in 2020, accounting for 16% of total mortality [128] that year. Therefore, there is an increasing interest in finding technological assistance solutions for the diagnosis and prediction of cancer.

In this context, Alexander Chowdhury et al. [129] conducted a comprehensive literature review to identify the latest applications of federated learning for cancer research and clinical oncology analysis. Their study came up with several positive results that contribute to the understanding of the use of federated learning in cancer diagnosis. Their results showed that many studies have been conducted in this area, but only 56% of them were focused on cancer research, while the others used cancer datasets to benchmark a general method. The studies dedicated to cancer research are listed in Table 6 below:

### 3.3. Discussion

Federated learning is a method for training ML models using decentralized data residing on different devices or systems as opposed to a central server. In the field of disease diagnosis, FL could be used to train models on a huge, distributed dataset of patient data from different hospitals or clinics. This method allows information and knowledge to be shared between facilities while protecting the privacy and security of patient data. Using a larger, more diverse dataset also allows for more accurate and robust models. However, implementations of federated learning for disease prediction, particularly cardiovascular disease, diabetes, and cancer, can be discussed from several perspectives, which are discussed in more detail in this section.

#### 3.3.1. Models Performance: Competition between FL and ML

In classical ML, data collection is the first step in the execution of the known pipeline. It is also known that the accuracy of a trained ML model can be improved by collecting additional data. Therefore, it is agreed in theory that the accuracy of FL models will surpass that of traditional ML models because FL can access more data due to its nature.

In this context, the prediction results presented in Table 4 using FL show the high feasibility and accuracy. For example, the models in [110] achieved 98.9% accuracy in detecting cardiac arrhythmias, whereas the models in [108] had 87.85% accuracy. In addition, both models in [103,112] had area under the curve values of 0.78 and 0.89, respectively. However, these results are not better than any classical ML models used to predict CVDs. Even though the results of [110] are relatively high, a comparison between other implementations and classical implementations shows that the accuracy of the classical ML is higher. For example, the machine learning models proposed in [102] achieved over 91% accuracy in predicting CVDs 12 months before their onset. These results outperform all FL implementations in Table 4 except [110].

On the other hand, the FL implementations in diabetes diagnosis showed relatively high performance values, with the authors in [118] recording an accuracy of 99.24%, which is better than the traditional ML models used in this field, as explained by the authors. Moreover, in [116,117], the authors stated that the results obtained were comparable to those obtained with traditional DL models. However, the results in [119] are not as high as those obtained with other implementations, with an accuracy of 72%, which is lower than the results obtained with conventional ML models, as shown in [35].

Furthermore, the results presented in Table 6 were inconsistent in comparing the performance between FL and the classical ML and DL models. In this regard, the results obtained in [132,134,136,136] proved that the FL and ML models (including the classical ML and DL) have the same performance. However, the results obtained in [130,138,139,141] proved that the FL models outperform the earlier implementations of ML. In contrast, the authors of the results in [144] clearly stated that the models of DL outperform the models of FL, in contrast to the results in [143] where the authors stated that the models of FL have higher generalizability than the models of ML, but not higher accuracy.

In summary, although FL may theoretically have higher performance in machine learning, the results obtained are not yet sufficient to prove this hypothesis in the field of disease prediction. The FL implementations in this field are very accurate and feasible, but in some cases, the models of ML are still able to provide higher accuracy even if privacy is not preserved.

#### 3.3.2. Real World vs. Research Implementations

Federated ML was proposed by Google in 2016 [30]. Although FL is still in its infancy, it has found widespread application in research, particularly in disease prediction.

However, most of the implementations performed, whether these were for cardiovascular diseases, diabetes, or cancer prediction, have been implemented as research studies rather than production methods. Moreover, most of these implementations are performed with publicly available data rather than using clinical or real-world data. For example, in the case of cardiovascular disease prediction, only [103] used real-world data from healthcare institutions and in the study in [104], real-world data from 10 individuals were used, whereas the others used either publicly available datasets or unspecified private data. In addition, none of these implementations were carried through to production readiness, but were conducted only as research studies.

In addition, the models for diabetes detection based on FL only used [121] data from a laboratory, whereas [118] used a dataset generated from a simulator and used other publicly available datasets. In addition, none of these implementations were taken to production maturity; all were conducted as research studies only. In contrast, for cancer detection, the studies in [139,142,143,144,145] used data from laboratories, whereas others used publicly available datasets, with the exception of [135,141], which used their own data without explaining their source. Similarly to the cardiovascular disease and diabetes cases, all studies were only research studies that were not production projects and were not made commercially available for further use. These findings support the fact that FL is still in its infancy and further efforts are needed to move into production phases with FL.

#### 3.3.3. Dedication to Disease Diagnosis

The implementations of federated machine learning that have been performed in the field of predicting diseases such as cardiovascular disease, diabetes, and cancer have not all directly been for diagnosing diseases. For example, in the prediction of cardiovascular diseases, all of the studies listed in Table 4 were aimed at proving privacy-preservation concepts. In addition, the studies in [103,104] attempted to solve scalability problems using CVDs, while [108] attempted to solve personalization nodes using FL, and [110] addressed explainability, where reducing communication costs contributed to both privacy and personalization. In this context, only [109] addressed the disease itself, without targeting other FL-related topics, because it used a dataset from a clinical laboratory.

In contrast, the diabetes FL-based implementations summarized in Table 3 were all devoted to the disease itself, without targeting other FL-related topics. The same is true for the studies listed in Table 4, as this table only includes FL-based models dealing with cancer, whereas the authors in [129] mentioned dozens of articles proposing some FL-based models trained with cancers but focusing on FL-related topics.

FL-based models are therefore able to analyze data from different institutions that are not connected or related in the real world, using specific disease datasets while targeting other FL-related ideas such as scalability, communication costs, personalization, and so on. This may potentially help increase the efficiency and accuracy of intelligent models in predicting disease by giving them access to more data, while also helping to advance the field itself, clearly a win–win scenario for machine learning and health scientists.

#### 3.3.4. Use of Smart Wearables

Smart wearables are known to provide people with continuous, long-term, and real-time monitoring. For example, fitness trackers and smartwatches have the potential to play an important role in the early detection and management of various diseases such as cardiovascular disease [34], diabetes [35], or even fatigue detection in the workplace, as shown in [36]. These tools can continuously monitor health data, such as the heart rate, and provide data that can help identify potential health problems. They also allow data to be collected outside of traditional healthcare settings, such as doctors’ offices and hospitals, so that a larger number of patients can be cared for over longer periods of time. Overall, the use of smart wearables can lead to the earlier diagnosis and treatment of diseases, improving outcomes and reducing healthcare costs.

The importance of smart wearables stems from their specifications, which have resulted from improvements in information and communication technologies (ICTs), the Internet of Things (IoT), and artificial intelligence. Smart wearables, as seen in [34,35,36], can be known as:

- Non-invasive: do not penetrate the skin to collect data;
- Compact: should not be bulky or large so as not to interfere with life activities;
- Affordable: to increase its acceptance;
- Rugged: to withstand harsh operating conditions such as light scratches or shocks;
- Easy to use: should have an intuitive interface;
- Durable power source: able to operate for a long period of time.

Despite the potential and usefulness of using smart wearables for disease detection using federated machine learning models, only one study ([104]) has employed a smart wearable to predict the onset of cardiovascular disease using data collected from a smartwatch for continuous, long-term, and real-time monitoring. In the other studies on cardiovascular disease, diabetes, or cancer, the use of smartwatches was not considered in the research. Therefore, there is still a great opportunity to merge smart wearables with the field of federated machine learning to enable private and secure model training without sharing confidential data.

#### 3.3.5. Limitations in the Use of FL for Disease Prediction

In this sense, the use of federated machine learning in the detection and prediction of CVDs, diabetes and cancer is still in its early stages. In addition to the fact that not all FL implementations beat classical ML models, very rare real-world examples in this context can be obtained. In addition, it is also rarely seen that FL researchers used smart wearables in their experiments. All these details are mentioned in Table 7 below, which summarizes the results discussed in this section to provide a complete overview of how implementations based on FL have contributed to different concepts. Moreover, other limitations and challenges that are obtained in the field of FL and its implementations in disease prediction are mentioned in Section 4.1 below.

## 4. FL in Disease Prediction: Challenges and Future Perspectives

Federated learning, the new and emerging technology, is promising and has already proven its efficiency in improving ML algorithms without compromising privacy. However, this technology still faces many challenges that require further research, which requires further development and improvement in this technology so that it can be further implemented in real-world scenarios. These challenges require further future work to bring this technology to a higher level so that it becomes more flexible and useful, contributing to its adoption in different areas of life. This section discusses these challenges and identifies the corresponding future perspectives needed to overcome obstacles and develop FL. These challenges demand further future work to bring this technology to a higher level to make it more flexible and useful, contributing to its adoption in different areas of life. This section discusses these challenges and identifies the corresponding future perspectives needed to overcome obstacles and develop FL.

### 4.1. Challenges

Federated learning is still in its early stages and still faces some obstacles. However, there is no unified classification of these challenges in the literature, and they can be considered differently depending on their nature, causes, and possible solutions. In this section, the challenges have been studied in detail and classified into three main categories [41,43,45,46,48,49,65,146,147]:

- Data source-related challenges (parties embedded in FL):
  - Structural heterogeneity;
  - Statistical heterogeneity;
  - Data specifications—amount and readiness.
- Learning process-related challenges:
  - Privacy;
  - High communication cost;
  - Aggregation techniques;
  - Personalization techniques;
  - Evaluation complexity.
- Other vulnerability-related challenges:
  - Federated fairness;
  - Application areas.

#### 4.1.1. Data Source-Related Challenges

⋆ **Structural heterogeneity:** This is also referred to as system heterogeneity. Since federated learning mainly aims to deal with data scattered in different islands, called parties, these parties may differ in terms of network state, storage space, performance, and the processing capabilities of the devices containing the parties’ data. Therefore, due to network failures, not all devices may be ready and online at each processing iteration, which is known as device failure. On the other hand, devices with better-processing capabilities train faster than other devices, resulting in unbalanced training times. Therefore, device failure and unbalanced training times can cause some devices to lag behind the global model if they are still training with outdated parameters, with these devices being referred to as laggards.
⋆ **Statistical heterogeneity:** Due to the differences between FL embedded parties, the data generated and collected are generally not independently and identically distributed (non-IID). Moreover, the data sizes of the different parties can be very different, resulting in an unbalanced distribution. This definitely increases the complexity in terms of analysis, modeling and evaluation.
⋆ **Data specifications—amount and readiness:** In classical machine learning and deep learning, the amount of training data is one of the factors affecting the performance of the models, where large amounts of data can increase the accuracy of the learned model. However, in a distributed environment, the amount of data on each party is not the same, and it may be insufficient for local training on some parties, which therefore affects the accuracy. In addition, heterogeneous data on the parties may require different preprocessing steps, where some parties can process some missing data while others do not.

#### 4.1.2. Learning Process-Related Challenges

⋆ **Privacy:** Despite the fact that federated learning aims to building smart models that do not collect user data, it is still vulnerable to data leakage caused by attacks. This is possible because of the transmission of gradients and partial parameters, whether this is between parties and manager in the centralized architecture or between parties themselves in the decentralized architecture. Those parameters are under the risk of cracking on three levels: the inputs, learning process, or learned model, as previously discussed. Usually, attacks are performed by adversaries ranging from malicious clients in a party to a malicious party which only has black-box access to the model. The types of attacks can be summarized into the following groups [54]:

- Poisoning attacks: these are conducted by injecting noise into the FL system, and are also split into two categories:
  - Data poisoning attacks: these are the most common attacks against ML models and can be either targeted toward a specific class or non-targeted. In a targeted attack, the noisy records of a specific target class are injected into local data so that the learned model will act badly on this class;
  - Model poisoning attacks: these are similar to data poisoning attacks, where the adversary tries to poison the local models instead of the local data.
- Inference attacks: in some scenarios, it is possible to infer, conclude, or restore the party local data from the model updates during the learning process;
- Backdoor attacks: secure averaging allows parties to be anonymous during the model update process. Using the same functionality, a party or group of parties can introduce backdoor functionality in in FL global model. Then, a malicious entity can use the backdoor to mislabel certain tasks such as choosing a specific label for a data instance with specific characteristics. For sure, the proportion of the compromised devices and FL model capacity affects in the intensity of such attacks.

⋆ **High communication cost:** this is induced by the huge number of involved devices, encryption and privacy preserving computations, local models and parameter-exchange batches. In addition, it is known that the life cycle of modern data is short and that the speed of iterative updating of data is fast, because the most important advantage is timeliness. Therefore, the cost of communication is a difficult topic that is worth studying;
⋆ **Aggregation techniques:** in centralized federated learning, the local models are aggregated into a global model at the central server. Due to the variety of amounts of data at each party, different results of local models, communication bottlenecks and other challenges, the method behind aggregating the global model is a challenging topic. In addition, most of the existing aggregation algorithms target the aggregation itself, communication/computation cost reduction or heterogeneity the most, while other topics such as personalization and scalability are less investigated;
⋆ **Personalization:** According to [148], there is a gap between the accuracy of local and global models, which impose personalization as a challenging topic in FL. However, there are no clear metrics to evaluate the performance of personalization techniques, which should be a hot topic for further research;
⋆ **Evaluation complexity:** In classical ML and DL, the models are evaluated by defined metrics such as accuracy, communication cost, computation speed, among others. In contrast, the evaluation of an FL system will add more parameters to be evaluated such as privacy, additional communication cost, and robustness against attacks.

#### 4.1.3. Other Vulnerabilities

⋆ **Federated fairness:** fairness is an emerging area of ML, investigating how to confirm that the results of a model do not depend on sensitive attributes in a way that is considered unfair. FL creates new problems for researchers regarding fairness and requires a greater focus on improving the fairness of existing algorithms. At present, it is unclear whether existing fairness methods and frameworks that have been shown to be effective in ML will also be effective in FL;
⋆ **Application areas:** federated learning has mainly been applied to supervised learning algorithms. Therefore, when using FL in domains that require data exploration, such as reinforcement learning, unsupervised learning, semi-supervised learning, and others, some challenges may arise;
⋆ **User adoption:** one of the main obstacles to integrating federated machine learning into disease diagnosis is user acceptance, adoption, and participation. Although FL is known as a privacy-friendly technology, FL is still new and has mixed user adoption due to privacy concerns, discomfort, ethics, and other contextual factors.

**Figure 8:**
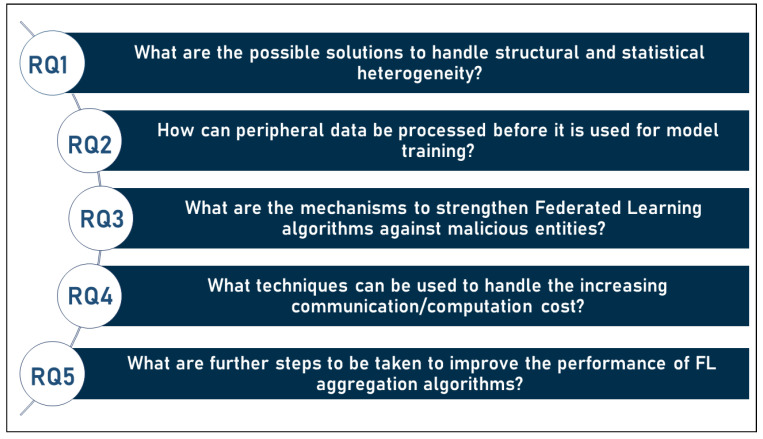
Research questions arising from analyzing the usage of FL in disease prediction.

Therefore, these difficulties give rise to the study questions below. In addition, these questions are illustrated in Figure 8 below (the initialism RQ in the list below and in the figure refers to the term “research question”):

- **RQ1:** Heterogeneity has a negative impact on the performance of a federated learning system. What are the solutions to deal with diversity?
- **RQ2:** Real-world data are noisy and usually not suitable for analysis by intelligent models. How can peripheral data be processed before these are used for model training?
- **RQ3:** Federated machine learning is vulnerable to security breaches and attacks. What mechanisms are in place to strengthen these algorithms against malicious entities?
- **RQ4:** The additional computations and sharing of models incur additional communication and computational costs in the FL system. What techniques can be used to manage the increasing costs?
- **RQ5:** The available aggregation algorithms consider aggregation, reduction in communication and computational costs, and privacy the most, while other issues such as personalization and scalability are the least considered. What further steps need to be taken to improve the performance of FL’s aggregation algorithms?

### 4.2. Future Perspectives

Federated learning technology is still in its infancy, and there is much room for improvement and enhancement that can increase its efficiency and feasibility. Based on the literature review and investigation of the major challenges in this area, the following future prospects can be identified in FL [41,43,45,46,48,49,65,146,149]:

⋆ **Managing heterogeneity:** Heterogeneity in federated learning systems can result from both data and hardware, which is known as statistical or structural heterogeneity. To overcome heterogeneity, federated learning researchers may consider the following:

- Structural heterogeneity:
  - Fault tolerance: FL considers the impact of low participation in the training process to resist device failures by storing user updates in a trusted cache architecture to mitigate their unreliable impact on the global model;
  - Resource allocation: to solve resource scarcity, most of the previous work is devoted to properly allocating resources to heterogeneous devices.
- Statistical heterogeneity:
  - Data clustering: separating independent data into multiple clusters, then processing FL on each cluster, which is not suitable for training bulk data due to conversion overhead;
  - Modify local training mode: put cross-entropy loss into the transfer process and assign different local update times to each party in each processing iteration;
  - Meta learning [150]: Improve training on non-IID data by creating a small subset of data that are shared among all edge devices.

⋆ **Privacy preservation enhancement:** even though the main goal of FL is to preserve privacy by sharing the trained model between entities instead of raw data, the privacy preservation concept needs further enhancement, especially towards:

- Enhancing security mechanisms: by proposing new robust and feasible security mechanisms that are protected against data attacks and cracking;
- Verifying the returned model: most privacy preserving methods (FL) assume that the clients are reasonably honest. Although this is in line with training rules, curiosity in acquiring private data remains. Therefore, the returned model should be checked to determine whether it can be considered non-malicious.

⋆ **Communication optimization:** due to the system and structural heterogeneity, as well as the decentralized nature of FL, the research area of the communication cost reduction is a hot topic to attend to. There are plenty things to be considered in this area, such as:

- Gradient aggregation: it is worthwhile to introduce adaptive weighting for each party or an ML method to learn how to aggregate these gradients in an efficient way;
- Handle heterogeneity: efficiently handling heterogeneous data and devices will definitely reduce communication rounds;
- Novel models of asynchrony: in the environment of FL, there is a large variety of devices where the synchronous scheme can be easily disrupted. Therefore, it is better to use an asynchronous scheme that can handle this diversity, solve the communication delay problem, and avoid concurrent training with heterogeneous devices; Therefore, the development of asynchronous FL platforms is a possible area of study;
- One-/few-shot learning: to minimize communication costs, reducing the number of learning rounds could be a viable solution. Some researchers are exploring the possibility of training the local models with only one iteration and updating the global model accordingly.

⋆ **Performance optimization:** The trade-off between communication, performance, and privacy is an active research area in FL. Performance optimization can be achieved using various approaches, such as:

- Incentive mechanism: to encourage parties’ participation in the training process in a feasible way, it is important to encourage high-quality users to contribute to the process by granting them some rewards, while neglecting or rejecting untrustworthy users because the inconsistent quality of data provided by users;
- Handle party dropouts: as one of the biggest challenges in networks with a large number of devices, handling dropouts will reduce communication costs, especially related to delayed parties;
- Personalization: improving FL personalization is much needed by users and has far-reaching applications. Many involved data holders will prefer to receive more personalized models to better meet their needs.

⋆ **Toward unsupervised learning:** unsupervised data are a large part of the data available in real life, and unsupervised learning is an area of great interest around the world. Therefore, it is of great efficiency to move towards unsupervised learning models with FL;
⋆ **Production of FL:** due to its novelty and lack of popularity, FL still needs to be put into production so that it can gain trust and be used in more areas of life;
⋆ **Benchmarks:** since the technology is still in its infancy, there is a large window of opportunity for benchmarking to define its future by ensuring that it is based on real-world circumstances, assumptions and datasets.

**Figure 9:**
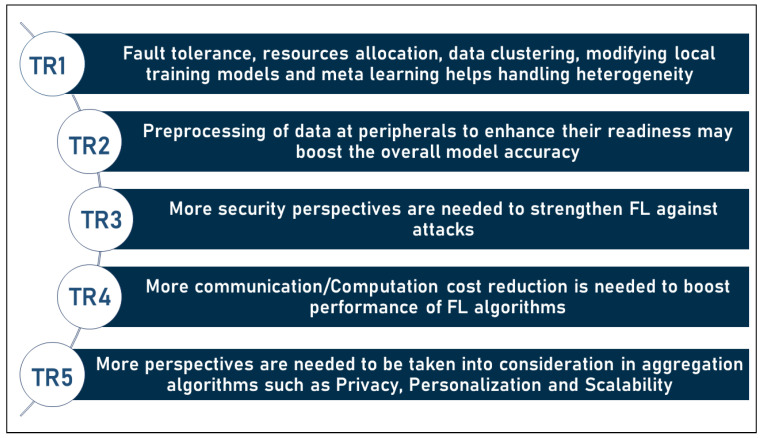
Research topics that may serve as solutions to the challenges in the domain.

For this reason, we can summarize the prospects on the following emerging research topics. Moreover, these research topics are shown in Figure 9 as follows (the symbol TR in the list below and in Figure refers to the term “trending research topic”):

- **TR1:** Fault tolerance, resource allocation, data clustering, modifying local training models, and meta learning help handle heterogeneity;
- **TR2:** Preprocessing of data at peripherals to enhance their readiness may boost the overall model accuracy;
- **TR3:** More security perspectives are needed to strengthen FL against attacks;
- **TR4:** More communication/computation cost reduction is needed to boost the performance of FL algorithms;
- **TR5:** more perspectives are needed to be taken into consideration in aggregation algorithms such as privacy, personalization, and scalability.

**Figure 10:**
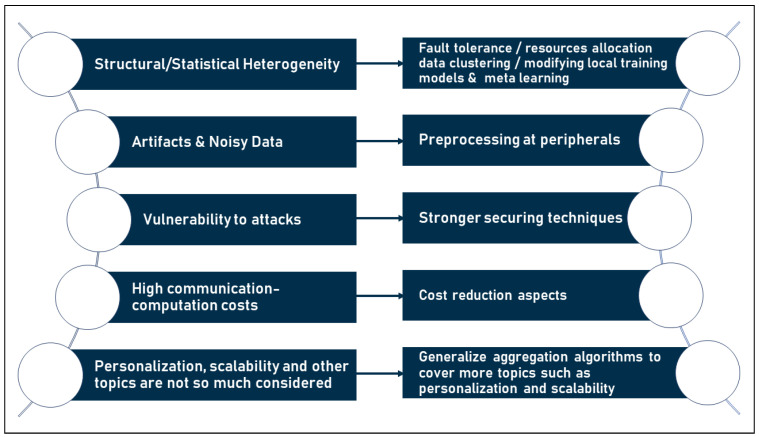
Challenges–future solutions chart.

Figure 10 below summarizes the challenges–future solutions relationship and illustrates how future views may act as potential solutions in the domain, all of which can assist in enhancing research on the use of federated machine learning in disease diagnosis and prediction.

## 5. Conclusions

It is hoped that the federated ML will solve the privacy problems of ML. It is attractive because it allows models to be trained without revealing sensitive information. Several aggregation strategies for FL knowledge have been proposed, although the field is still in its infancy. There are several examples of the application of this technology in various industries, including healthcare, banking, and others. As explained in this article, it has been used in healthcare as a diagnostic tool for a number of diseases, including cardiovascular disease, diabetes, and cancer. Federated machine learning has achieved some successes so far, but still faces challenges such as the diversity of data and devices in the FL network, the possibility of security breaches and attacks, and the high cost of computation and communication. To help future researchers understand where we are now with this technology and what they need to take the following steps, this article presents a number of future directions that could be pursued to address these obstacles and improve the efficiency of this technology.

## Figures and Tables

**Table 1:** Differences among FL groups divided by type of data.

	Horizontal Transfer Learning	Vertical Transfer Learning	Federated Transfer Learning
Data Distribution Similarity	Same	Different	Different
Output/Label Space Similarity	Different	Same	Same
Type of Task	Single task	Single ask	Federated task

**Table 2:** Summarized Taxonomy for Federated Learning Systems.

Taxonomy	Category	Structure	Advantages
**Data partitioning**	Horizontal FL	Different parties and similar data features	Holds larger variety of parties
	Vertical FL	Similar parties and different data features	Holds larger variety of data features
	Federated transfer learning	Different parties and different data features	Holds larger variety of parties and data features
**Machine learning models**	Linear models	Linear regression, ride regression, lasso regression	Ease of implementation
	Decision tree	gradient boosting, decision trees, random forests	Accurate, stable, and can map non-linear relationships
	Neural networks	-	Learning capabilities, highly robust and fault-tolerant
**Privacy mechanisms**	Model aggregation	Central manager learns by aggregating the locally trained model	Avoid transmitting original data
	Cryptographic methods	Using encryption algorithms such as homomorphic encryption and secure multi party computation (SMC) to encrypt the messages exchanged among parties	Enables the calculation and processing of encrypted data
	Differential privacy	Reducing the impact of a single data record on the calculation of the global model	Reduce the effect of data poisoning attacks
**Methods for solving heterogeneity**	Asynchronous communication sampling	To resolve the heterogeneity of parties	Solve the problem of communication delays and avoid simultaneous training with heterogeneity of parties
	Fault-tolerant mechanism	To resolve the failure of parties	Prevent whole system from collapsing if one of the parties failed
	Heterogeneous model	To resolve the heterogeneity of data	Resolve the issue of models diversity
**Communication architecture**	Centralized design	Architecture controlled by a central aggregation manager/server	Reduces communication cost
	Decentralized design	Communication performed among parties without the existence of a central manager/server	Reduces the risk of backdoor attacks
**Scale of federation**	Cross-silo FL	Parties are less in number, hold large amounts of data and equipped with high computation power	Fits for FL among institutions
	Cross-device FL	Parties are high in number, hold less amount of data and equipped with less computation power	Fits for FL among individuals
**Motivation of federation**	Regulations	Motivated by laws such as GDPR and others	
	Incentives	Motivated by desire of updating some services	Enhancing ML services

**Table 3:** Contributions of existing FL aggregation algorithms.

Ref#	Year	Given Name	Model Aggregation	Convergence Reduction	Heterogeneity	Security	Communication Cost	Computation Cost	Fault Tolerance	Learning Quality	Scalability	Personalization
[30]	2017	FedAVG	✓									
[66]	2017	-				✓	✓					
[67]	2019	RFA	✓			✓						
[68]	2020	SCAFFOLD	✓	✓			✓					
[69]	2020	FedOPTFedADAGARFedYOGIFedADAM	✓	✓	✓							
[70]	2020	FedBoost					✓					
[71]	2020	FedProx		✓	✓							
[72]	2020	FedMA	✓				✓					
[73]	2020	-	✓	✓			✓					
[74]	2020	-					✓	✓				
[75]	2020	-	✓				✓					
[76]	2020	LAQ					✓					
[77]	2020	SAFA	✓				✓	✓	✓			
[78]	2021	FedDist			✓							✓
[79]	2021	FEDHQ	✓	✓								
[80]	2021	FAIR	✓							✓		
[81]	2021	FedPSO					✓			✓		
[82]	2021	LEGATO				✓	✓	✓			✓	
[83]	2021	MHAT	✓		✓			✓				
[84]	2021	-				✓						
[85]	2021	-	✓		✓					✓		
[86]	2021	SEAR				✓		✓				
[87]	2021	Turbo-Aggregate	✓					✓			✓	
[88]	2022	EPPDA				✓			✓			
[89]	2022	FedBuff	✓									
[90]	2022	HeteroSAg	✓			✓	✓					
[91]	2022	LightSecAgg						✓	✓			

**Table 4:** Federated machine learning implementations in CVDs prediction.

Ref	Year	Type	Parameter Studied	Predicted outcome	Model	FL Architecture	Contribution	Dataset Used	Performance
	2018	Classification	Electronic health records	Hospitalization for CVD patients	Federated optimization scheme (cPDS) for solving sparse support vector machine		Scalability Privacy	Electronic heart records from the Boston Medical Center	Best 0.78 AUC
	2020	Regression	Heart rate	Heart rate	Federated; earning based on sequential Bayesian method (FD Seq Bayes) Empirical Bayes-based hierarchical Bayesian method (FD HBayes-EB)	Centralized Decentralized	Privacy Scalability	Private	-
	2021	Regression	Blood pressure	Blood pressure	Time-series-to-time-series generative adversarial network (T2T-GAN) (based on LSTM)	Centralized	Novelty Privacy	Cuff-Less blood pressure estimation [106] University of Queensland vital signs dataset [107]	Mean error of 2.95 mmHg and a standard deviation of 19.33 mmHg
	2021	Classification	ECG	Arrythmias	Customized alignment Model	Centralized	Personalization Privacy	Private	Accuracy: 87.85%
	2021	Classification	Electronic health records	Cardiovascular risk	Sequential pattern mining (SPM) Based Framework	Centralized Decentralized	Privacy	Nursing Electronic Learning Laboratory (NeLL)	-
	2022	Classification	ECG	Arrythmias	1D-convolutional neural Networks	Centralized	Privacy Explainability Communication cost reduction Personalization	MIT-BIH arrhythmia Database [111]	Accuracy: 98:9%
	2022	Classification	Cardiovascular magnetic resonance images	Hypertrophic cardiomyopathy	3D-convolutional neural networks	Centralized	Privacy	M&M challenge [113] ACDC challenge [114]	Best 0.89 AUC

**Table 5:** Federated machine learning implementations in diabetes prediction.

Ref	Model	Data Used	Performance
[116]	FL deep neural network	SFU prototype swept-source OCTA RTVue XR Avanti (OptoVue, Inc.) Angioplex (Carl Zeiss Meditec) PLEX Elite 9000 (Carl Zeiss Meditec)	Performance is comparable to conventional DL models
[117]	Not identified	Health Facts EMR Data dataset from Cerner	Performance is similar to the gold standard of centralized learning
[118]	FL convolutional neural network (CNN) FL multilayer perceptron (MLP)	Generated by simulator	FL-CNN recall: 99.24% FL-MLP recall: 98.69% performed better than traditional DL
[119]	Not identified	Fine-Grained Annotated Diabetic Retinopathy (FGADR) dataset [120]	Accuracy: 72%
[121]	Not identified	Private data collected from different healthcare facilities	-
[122]	Standard FL FedAVG FedProx	EyePACS [123] Messidor [124] IDRID [125] APTOS [126] University of Auckland (UoA) [127]	Standard FL Accuracy: 92.19% FedAVG Accuracy: 90.07% FedProx Accuracy: 85.81%

**Table 6:** Federated machine learning implementations in cancer prediction.

Ref	Disease	Data Used	Performance
[130]	Brain tumor	Brain MRI Segmentation Kaggle dataset [131]	FL results outperform the baseline but classical ML models competed with their results
[132]	Brain tumor	BraTS dataset [133]	Dice = 0.86 for both FL and ML scenarios
[134]	Brain tumor	BraTS dataset [133]	FL performance is similar to ML models
[135]	Brain tumor	Private data	Dice=0.86 for both FL and ML scenarios
[136]	Skin cancer	ISIC 2018 dataset [137]	Accuracy = 91% for both FL and ML scenarios
[138]	Skin cancer	ISIC 2019 Dermoscopy dataset [137]	Accuracy: 89% which outperformed previous implementations
[139]	Breast cancer	Private data from 7 different institutions	FL perform 6.3% on average better than classical ML
[140]	Breast cancer	Obtained from Netherlands Cancer Registry (NCR)	Not available
[141]	Prostate cancer	Private data	FL model exhibited superior performance and generalizability to the ML models
[142]	Lung cancer	Private data from 8 institutes across 5 countries	Not available
[143]	Pancreatic cancer	Data from hospitals in Japan and Taiwan	FL models have higher generalizability than ML models
[144]	Thyroid cancer	Private data from 6 institutions	DL models outperformed FL models
[145]	Anal cancer	Private data from 3 institutions	Not available

**Table 7:** Federated machine learning implementations in CVDs prediction.

Ref	Disease	FL Beats ML *(Performance)*	Real-World Implementation	Disease-Oriented	Wearable
[103]	CVDs	No	No	No	No
[104]		No	Yes	No	Yes
[105]		No	No	No	No
[108]		No	No	No	No
[109]		No	No	Yes	No
[110]		Yes	No	No	No
[112]		No	No	No	No
[116]	Diabetes	Yes	No	Yes	No
[117]		Same	No	Yes	No
[118]		Yes	No	Yes	No
[119]		No	No	Yes	No
[121]		Not available	No	Yes	No
[122]		Yes	No	Yes	No
[130]	Cancer	Yes	No	Yes	No
[132]		Same	No	Yes	No
[134]		Same	No	Yes	No
[135]		Same	No	Yes	No
[136]		Same	No	Yes	No
[138]		Yes	No	Yes	No
[139]		Yes	No	Yes	No
[140]		Not available	No	Yes	No
[141]		Yes	No	Yes	No
[142]		Not available	No	Yes	No
[143]		No	No	Yes	No
[144]		No	No	Yes	No
[145]		Not available	No	Yes	No

## Data Availability

The study did not report any data.
